# Impact of vitamin B12 on the reproductive health of women with sickle cell disease: a narrative review

**DOI:** 10.1530/RAF-23-0015

**Published:** 2023-07-20

**Authors:** Tarimoboere Agbalalah, Faith Owabhel Robert, Emmanuel Amabebe

**Affiliations:** 1 Department of Anatomy, Faculty of Basic Medical Sciences, Baze University, Abuja, Nigeria; 2 Department of Medical Biotechnology, National Biotechnology Development Agency, Abuja, Nigeria; 3 Department of Medical Biochemistry, Rivers State University, Port Harcourt, Nigeria; 4 Department of Oncology and Metabolism, University of Sheffield, Sheffield, UK

**Keywords:** sickle cell disease, vitamin B12, reproductive health, pregnancy, homocysteine

## Abstract

Sickle cell disease (SCD) poses an increased risk of infertility, pregnancy complications and maternal and perinatal mortality among women of reproductive age. This risk is particularly higher for women in sub-Saharan Africa, where the disease burden is highest and access to comprehensive health care is limited, as well as in other countries with a high SCD prevalence due to migration. Disease-modifying treatments for SCD could directly and indirectly harm the ovaries, potentially compromising the quality and quantity of existing oocytes. Therefore, it is essential to explore alternative interventions, such as nutritional modifications that are less harmful and cost-effective in order to improve reproductive outcomes and enhance the overall well-being of both mother and child in this population. Maintaining optimal levels of B12 may possibly provide benefits to the ovaries and pregnancy by decreasing homocysteine levels, increasing nitric oxide (NO) bioavailability and promoting antioxidant and anti-inflammatory activities. Individuals living with SCD are more susceptible to vitamin B12 (B12) deficiency. However, there is a lack of clinical data investigating the relationship between systemic levels of B12, its supplementation, and reproductive outcome measures in SCD women. Therefore, this review aims to examine the current evidence regarding the impact of SCD on female reproductive health and the role of B12 in the reproductive biology of women living with SCD.

**Lay summary:**

Poor reproductive health is a concern for patients with sickle cell disease (SCD). SCD can lead to damage to the ovaries. Most of the therapies for sickle cell disease are toxic to the ovaries, expensive and unavailable to affected women, particularly those living in resource-poor areas such as sub-Saharan Africa. There is a need to seek less harmful and affordable interventions such as nutrition to improve reproductive outcomes for women with SCD who are of child-bearing age. Good levels of vitamin B12 have been found to maintain ovarian health and pregnancy. Patients with SCD have been reported to have a high risk of vitamin B12 deficiency, but the impact of B12 on reproduction in this group of women is yet to be evaluated. The review explores current evidence of the impact of both SCD and B12 on female reproduction.

## Introduction

Sickle cell disease (SCD) refers to haemoglobinopathies consisting of at least one haemoglobin (Hb) S allele expressed as homozygous (HbS/S, most common and fatal), heterozygous (HbS/C, which is less severe), two phenotypes of sickle beta (β) thalassemia (HbS/β^+^ thalassemia and HbS/β^o_^thalassemia) and other rare forms such as HbS/D, HbS/O and HbS/E (Serjeant & Vichinsky 2018). SCD is the most common monogenic and autosomal recessive disorder caused by a missense variant (rs334) in the Hb subunit β-globin (HBβ) gene, which results in morphological abnormalities in the red blood cells (RBC) (Serjeant & Vichinsky 2018). The morphological abnormalities in the RBC results in haemolysis and/or vaso-occlusion, leading to delayed growth and sexual maturation, as well as progressive damage to most organs including the bones, ovaries, brain, kidneys, lungs and more (Meremikwu & Okomo 2016). Thus, SCD has a negative impact on the quality of life of affected individuals, particularly those with limited access to comprehensive team care and the high cost of care.

SCD is a global public health burden. It is highly prevalent in malaria-endemic regions and commonly encountered in Africans, Mediterraneans and South Asians. Africa has the highest prevalence rates, with 20–30% in countries such as Nigeria, Tanzania, Republic of Congo, Cameroon, Gabon and Ghana (Grosse *et al.* 2011). SCD prevalence is steadily increasing in Europe, USA and the UK due to migration (Roberts & Montalembert 2007). There is high nutritional demand to sustain normal physiologic functions in SCD due to increased basal metabolic rate and constant erythropoiesis. Consequently, SCD is characterised by both macro- and micronutrient deficiencies (Mandese *et al.* 2016). Among the micronutrient deficiencies, vitamin B12 (B12) deficiency is common in individuals with SCD, regardless of age, and it has both haematological and reproductive consequences. Therefore, low circulating levels of B12 may not only worsen the clinical manifestations of SCD but also lead to unfavourable reproductive outcomes by impacting fertility in women living with SCD (Gaskins *et al.* 2015).

Given the beneficial role of optimal B12 levels in females, including improving reproductive outcome in both natural and assisted pregnancies (Gaskins *et al.* 2015), it is important to explore the impact of B12 in SCD women of child-bearing age. This is particularly crucial in sub-Saharan African countries, which bear the highest burden of SCD and often have limited access to comprehensive health care, as well as in other countries of the world with a high prevalence of SCD. Therefore, this review aims to examine the current knowledge on the impact of SCD on female reproductive health and explore the role of B12 on the reproductive biology of SCD women.

## Literature search

A comprehensive literature search was performed from June 2022 to November 2022 using PubMed, MEDLINE and Google Scholar databases. Multiple search terms were employed, including vitamin B12, SCD, the global prevalence of SCD and the impact of SCD on reproductive health. Additionally, specific searches were conducted to explore the effect of vitamin B12 on the reproductive health of women with SCD and the consequences of vitamin B12 deficiency on female infertility, fertility and pregnancy outcomes, as well as potential mechanisms contributing to poor reproductive outcomes in SCD. No restrictions were placed on publication dates, and only articles written in English were included.

## Influence of SCD on female reproductive health

**Figure 1:**
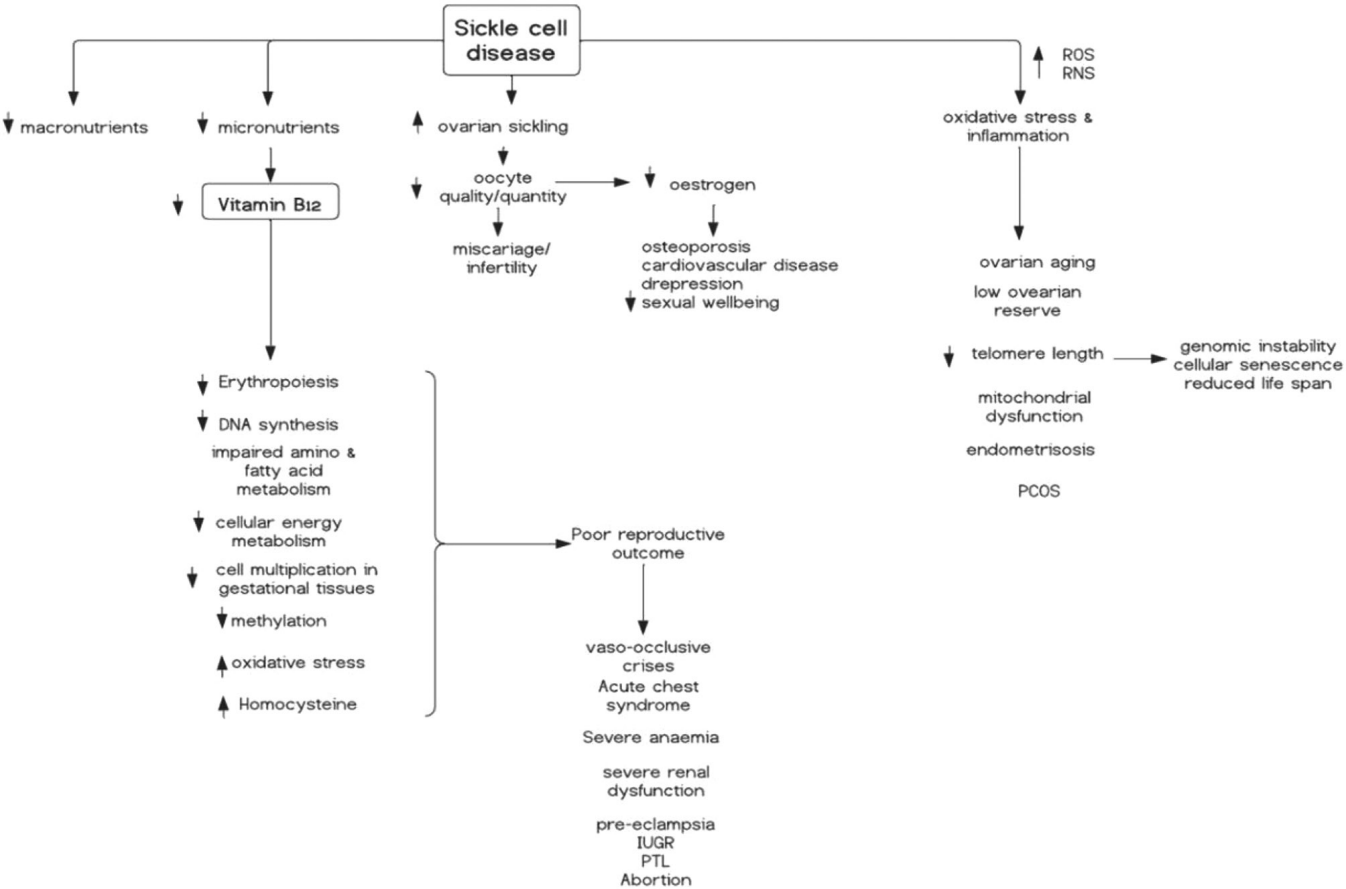
Impact of sickle cell disease (SCD) on female reproductive health. Sickle cell disease appears to reduce circulating vitamin B12 levels leading to decreased erythropoiesis, DNA synthesis and methylation, cell multiplication in gestational tissues, and increased oxidative stress and inflammation, as well as homocysteine accumulation. SCD-induced oxidative damage and inflammatory imbalance in the ovaries can cause ovarian aging, low ovarian reserve and decreased telomere length of oocytes, mitochondrial dysfunction, endometriosis and polycystic ovarian syndrome (PCOS). These can lead to other adverse reproductive outcomes such as IUGR and PTL. Additionally, SCD may negatively impact the ovaries by increasing ovarian sickling and reducing ovarian reserve, resulting in decreased oestrogen which increases the risk of osteoporosis and cardiovascular disease whilst decreasing sexual well-being. IUGR, intrauterine growth restriction; PTL, preterm labour; ROS, reactive oxygen species; RNS, reactive nitrogen species. Figure created in Lucidchart.

SCD has a negative impact on the reproductive health of both men and women. In women, the disease significantly influences the ovaries (which are vital for maintaining female reproductive potential and endocrine stability), leading to a reduced reproductive lifespan (Ghafuri *et al.* 2017). The negative impact on the ovaries affects ovarian follicular dynamics and ovulation , increases ovarian sickling, causing ischemia and reperfusion injury to the ovaries (Ghafuri *et al.* 2017), and impair oocyte quality and quantity which increases the risk of infertility (Fig. 1) (Kopeika *et al.* 2019, Garba *et al.* 2021).

## Gonadal hypofunction

Gonadal hypofunction has been reported in females with SCD (Gardner *et al.* 2016). It has been suggested that chronic transfusion and haemochromatosis in females with severe SCD may be associated with low ovarian reserve, a gradual decline in ovarian follicle number and quality, which may be a causative factor for infertility and failure in assisted reproductive technology (ART) (Chang *et al.* 2018). Additionally, ovarian sickling resulting from frequent intravascular sickling, vascular occlusion, infarction and the release of inflammatory mediators during ischemia, as well as recurrent ovarian hypoxia, could also contribute to ovarian dysgenesis and premature ovarian failure in women with SCD (Chase *et al.* 2009). It is worth noting that frequent ovarian hypoxia can rapidly deplete ovarian reserve compared to cases without chronic hypoxic injury to ovarian tissues (Chase *et al.* 2009).

In addition to causing miscarriage and infertility, women with a reduced ovarian reserve are more susceptible to diseases characterised by decreased oestrogen, such as osteoporosis, increased cardiovascular morbidity and mortality, depression and diminished sexual well-being (Chang *et al.* 2018). Few studies have evaluated the ovarian reserve, an indicator of the female reproductive potential, in SCD women. In a cross-sectional study of 166 participants (83 HbSS and 83 HbAA women), it was observed that serum anti-Müllerian hormone (AMH) levels in HbSS women were two times lower than those in HbAA women. Additionally, there was diminished ovarian reserve in HbSS women compared to age-matched HbAA women (Garba *et al.* 2021). Another study, based on 285 banked serum samples from 93 HbSS women, of which 86 out of 93 were exposed to hydroxyurea (a disease-modifying therapy for SCD that reduces the frequency of vaso-occlusive crises, and need for blood transfusion), found a reduction in serum AMH concentrations. These concentrations were lower than the median levels observed in age- and sex-matched reference values (Pecker *et al.* 2020). Additionally, Kopeika *et al.* discovered significantly low levels of AMH in 50 heterozygous SCD subjects, of which 8 out of 50 were exposed to hydroxyurea, when compared to 73 age- and ethnicity-matched controls without haemoglobinopathy (Kopeika *et al.* 2019). More so, the study found that serum AMH declined more rapidly in women with SCD than in controls starting from the age of 30 (Kopeika *et al.* 2019). Disease-modifying treatments for SCD, such as hydroxyurea and chronic blood transfusion, may indirectly or directly damage the ovaries and compromise existing oocyte quantity or quality (Pecker *et al.* 2020).

## Pregnancy complications

Pregnancy in women with SCD results in an increased incidence of maternal and perinatal complications, such as pre-eclampsia, preterm labour, intrauterine growth restriction (IUGR), and abortions (Sousa *et al.* 2022). Additionally, pregnancy aggravates the pre-existing anaemia in women with SCD, leading to an increased incidence of severe anaemia and frequent need for blood transfusion (Smith-Whitley 2019). The hematologic changes that occur during pregnancy could place an excessive burden on organs already affected by chronic injuries resulting from SCD (Silva-Pinto *et al.* 2014). Anaemia in SCD pregnancy prompts defective placental perfusion, which reduces nutritional substrate transport and oxygen transfer to the foetus, leading to an increased incidence of IUGR in pregnancies affected by SCD (Smith-Whitley 2019).

The cardiovascular and respiratory adaptations in SCD pregnancy pose significant challenges and can be life threatening. These adaptations result in increased cardiopulmonary demands, particularly in women with SCD-induced pulmonary hypertension (Baptista *et al.* 2016). Pregnant SCD patients experience increased metabolic demand, and blood viscosity, as well as hyper-coagulability, which are associated with a high incidence of vaso-occlusive crises, acute chest syndrome, osteonecrosis, hepatic necrosis and thromboembolic events (Villers *et al.* 2008). Vaso-occlusion also occurs in the placenta, leading to villous fibrosis, necrosis and infarction. This compromises uteroplacental circulation, resulting in chronic foetal hypoxia and adverse foetal outcomes (Villers *et al.* 2008).

## Oxidative stress and inflammation as potential mechanisms for poor reproductive outcomes in SCD

The negative impact of SCD on the ovaries and pregnancy can be attributed to oxidative stress (OS) and inflammation (Conran & Belcher 2018). SCD is characterised by a high level of reactive oxygen species (ROS) and reactive nitrogen species (RNS) production, leading to OS. This, in turn, promotes an inflammatory imbalance in this population (Nader *et al.* 2018). Pathological events in SCD, including frequent polymerisation of HbS, the release of haem and iron from haemolysed RBC and the decreased nitric oxide (NO) bioavailability in the vascular compartment contribute to the formation of ROS and RNS and subsequently OS (Nader *et al.* 2018).

ROS are important modulators of ovarian germ cell and stromal cell physiology, playing significant and diverse roles in reproductive biology (Zhang *et al.* 2016). Normal ROS levels modulate various signalling transduction pathways in folliculogenesis, oocyte maturation, ovulation, blastocyst formation, fertilisation, implantation, luteolysis, and feto-placental development (Kala & Nivsarkar 2016). However, excessive ROS production may possibly cause oxidative damage to the ovaries by promoting lipid peroxidation cascades, which influence folliculogenesis and ovulation and can lead to ovarian aging or low ovarian reserve (Maclaran & Nikolaou 2019). OS stimulates telomere shortening, mitochondrial dysfunction, inflammation and granulosa cell (GC) apoptosis, reducing communication between oocytes and GCs and affecting pre-ovulatory oocyte maturation. It also accelerates corpus luteum degeneration (Cajas *et al.* 2020).

Telomeres, the nucleoprotein-DNA structures that maintain genome integrity and chromosome stability, can shorten due to oxidative damage to the ovaries. This process promotes genomic instability, cellular senescence and reduced lifespan (Smith *et al.* 2020). It is worth noting that telomere length in cumulus cells has been found to be positively correlated with oocyte and embryo quality (Cheng *et al.* 2013). In a study by Xu *et al.,* an association was observed between primary ovarian insufficiency (POI) and shortened telomeres, as well as diminished telomerase activity in granulosa cells (Xu *et al.* 2017).

The mitochondria, which supply energy and modulate cellular signalling for oocyte maturation, fertilisation, and embryogenesis through aerobic respiration, are linked to ovarian aging (Pasquariello *et al.* 2019). Ovarian aging is characterised by reduced oocyte mitochondrial DNA content (Pasquariello *et al.* 2019), and inhibiting adenosine triphosphate (ATP) synthase activity can prevent germ stem cells from developing into oocytes (Teixeira *et al.* 2015). An overview of the impact of SCD on the female reproductive health is shown in Fig. 1.

## Impact of vitamin B12 on reproductive health of women living with SCD

B12 (cobalamin) is synthesised solely by bacteria or archaea and is naturally present in foods of animal origin, seafood, milk and fortified cereals (Watanabe & Bito 2018). It is also available as a dietary supplement and a prescription medication (Watanabe & Bito 2018). B12 is required for nervous and reproductive systems, normal erythropoiesis and growth, DNA synthesis, amino and fatty acid metabolism, and cellular energy metabolism (Ge *et al.* 2022). Additionally, B12 maintains normal folate metabolism, which is essential for cell multiplication in rapidly dividing placental and foetal tissues (Mahajan *et al.* 2019). It participates in one-carbon metabolism cycle, thus, functioning in numerous methylation reactions that occur in developing embryos and regulates foetal growth (Mahajan *et al.* 2019).

**Figure 2:**
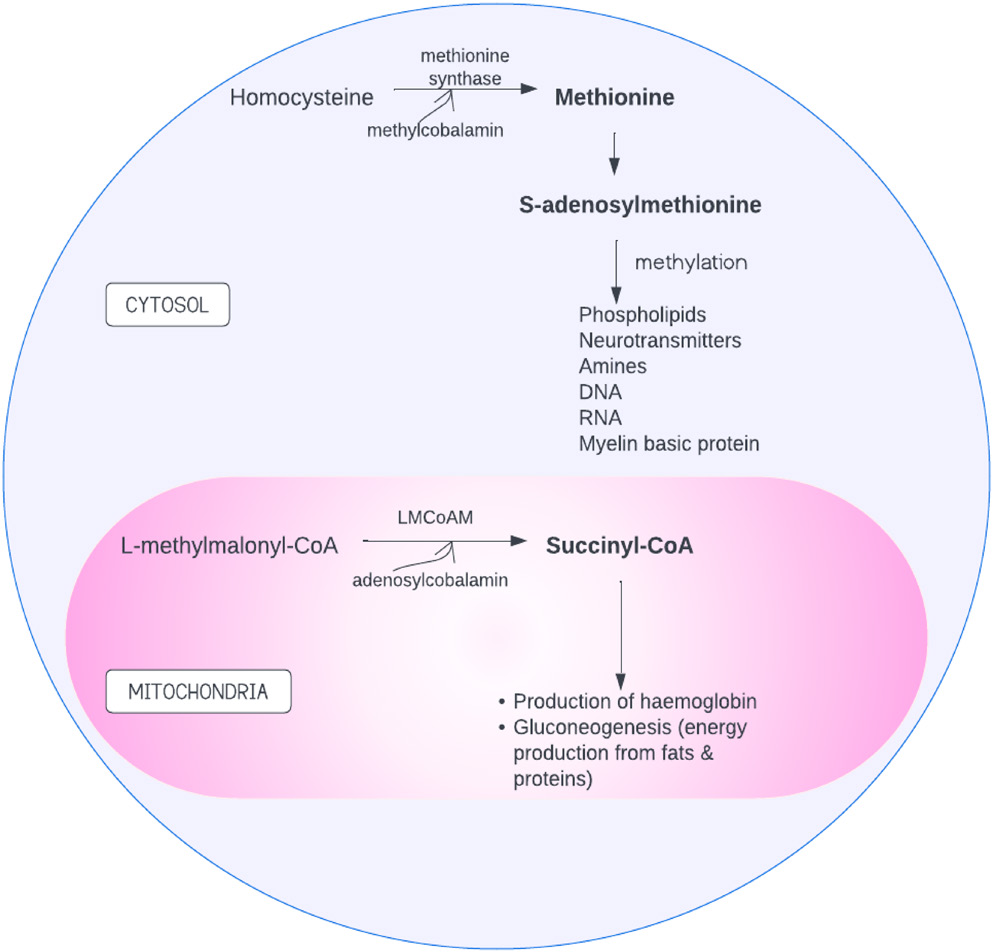
Formation of S-adenosyl-methionine and succinyl-CoA with vitamin B12 as coenzyme. Vitamin B12 facilitates the metabolism of homocysteine and l-methyl-malonyl-Co-A to prevent their build-up and deleterious effects of homocysteine.LMCoAM,l-methyl-malonyl-CoA mutase. Figure created in Lucidchart.

In humans, B12 functions as a coenzyme in two enzymatic reactions (Kräutler 2012). In the first enzymatic reaction, methyl-cobalamin acts as a coenzyme in the methionine synthase reaction, which converts homocysteine to methionine in the cytosol (Fig. 2). Methionine is an important precursor for the formation of S-adenosyl-methionine, which is essential for the methylation of phospholipids, neurotransmitters, amines, DNA, RNA and myelin basic protein (Fig. 2) (Froese *et al.* 2019). A decrease in S-adenosyl-methionine could lead to impaired DNA methylation, which may alter foetal metabolic programming and increase the risk of non-communicable diseases later in life (Randunu & Bertolo 2020). Therefore, a deficiency in B12 may result in homocysteine accumulation and decreased circulating methionine, affecting reproduction, protein and DNA methylation (Akamine *et al.* 2021).

In the second enzymatic reaction, adenosyl-cobalamin functions as a coenzyme in the conversion of l-methyl–malonyl-CoA to succinyl CoA in the mitochondria catalysed by l-methyl-malonyl-CoA mutase (Fig. 2) (Froese *et al.* 2019). This reaction plays a significant role in extracting energy from proteins and fats. Furthermore, succinyl CoA is necessary for the production of haemoglobin (Dowd *et al.* 1975). This suggest that insufficient B12 may lead to succinyl CoA deficiency, resulting in reduced gluconeogenesis and haem synthesis, which is capable of causing growth retardation and anaemia (Bicakci 2015).

## Vitamin B12 deficiency and female reproductive health

B12 plays a critical role in maintaining healthy reproduction by improving the function of reproductive organs, reducing OS, pro-inflammatory cytokines, and circulating homocysteine levels (Chan *et al.* 2018, Shakoor *et al.* 2021). Having optimal B12 levels, defined as serum B12 between 369 and 959 pmol/L (Dommisse 1991), at preconception is associated with favourable reproductive outcomes in both natural pregnancies and those after ART (Cirillo *et al.* 2021). Deficient levels of B12, characterised as serum B12 < 200 pmol/L, may have a significant impact on women’s reproductive health. This deficiency may lead to anovulation, disrupt normal cell division, impair egg development and result in difficulties during implantation (Bennett 2001). Insufficient B12 in pregnant women may contribute to various complications including preeclampsia, spontaneous abortion, IUGR, preterm labour, neural tube defects and increased perinatal morbidity and mortality. Insufficient B12 could also lead to poor foetal growth, which increases the risk of long-term health issues in the offspring, including a high susceptibility to non-communicable diseases (Gernand *et al.* 2016).

Demand for B12 increases during pregnancy due to rapid cell multiplication and the need for B12 transport to the foetus. As a result, there is a gradual physiological decline in maternal B12 levels, which could be worsened by factors such as haemodilution, hormonal fluctuations, impaired renal function, or altered concentration of binding proteins (transcobalamin and haptocorrin). The lowest concentration of B12 is typically observed during the third trimester, but it returns to pre-pregnancy levels within a few weeks postpartum (Behere *et al.* 2021). B12 deficiency may also intensify anaemia, which is the most common complication of pregnancy, as it leads to low concentrations of haemoglobin. This can lead to decreased foetal oxygenation and abnormal foetal outcomes.

B12 deficiency negatively impacts the reproductive health of women, but its effects on women living with SCD are not fully understood. Individuals with SCD are at risk of B12 deficiency (Ahmed *et al.* 2016), which may be attributed to the increased rate of haematopoiesis (van der Dijs *et al.* 1998). Pregnancy in SCD women is associated with a higher risk of maternal and foetal mortality (Alayed *et al.* 2014), necessitating expensive intensive care facilities and prolonged hospital stays. Therefore, it is crucial to explore cost-effective interventions, such as nutrition, to improve reproductive outcome and enhance the quality of life of women living with SCD. These nutritional interventions are particularly relevant in sub-Saharan African countries where the disease burden is higher, as well as in Europe, the USA and the UK, where prevalence is increasing due to migration.

## Infertility

B12 deficiency is a significant factor contributing to difficulties in conceiving. A study found a link between low levels of B12 and female infertility and subfertility when compared to fertile women (Isomah *et al.* 2021). As mentioned earlier, B12 is vital for homocysteine metabolism, and insufficient B12 levels leads to its accumulation. Consequently, B12 deficiency can indirectly contribute to infertility by increasing homocysteine levels. The accumulation of homocysteine promotes OS by inhibiting antioxidant enzymes, which could be harmful to oocytes and impair ovulatory function, leading to anovulation (Michels *et al.* 2017). Increased homocysteine levels could also damage the endometrium, resulting in defective implantation and chemical pregnancy, which refers to early pregnancy occurring shortly after implantation (before the sixth week) (Pacchiarotti *et al.* 2007). Many women with unexplained infertility have been found to have elevated serum homocysteine levels (Dubey *et al.* 2016). Although higher homocysteine levels have been reported in individuals with steady-state HbSS compared to age- and sex-matched HbAA controls, whether this is caused by B12 deficiency has not been determined (Uche *et al.* 2019).

## Fertility and pregnancy outcomes

Many women experiencing infertility have shown increased circulating homocysteine levels, which is characteristic of low B12, B6 and folate levels (Dubey *et al.* 2016). There is evidence indicating that B12 supplementation is linked to improved pregnancy outcome. In a retrospective study involving 269 Caucasian women undergoing ART, it was observed that women who received 5µg B12 daily had a higher likelihood of achieving clinical pregnancy and live birth (Cirillo *et al.* 2021). Additionally, improved fertility outcomes have been observed when homocysteine levels were reduced through interventions targeting B12, B6 and folate (D’Uva *et al.* 2007).

## Oocyte maturity

Increased homocysteine have been reported to negatively impact oocyte maturation, fertilisation and embryo quality, whereas reducing homocysteine has shown improvements in oocyte maturity and quality (Akamine *et al.* 2021). This is supported by a study that found an increased percentage of oocyte maturity and quality following a decrease in homocysteine concentration in the follicular fluid (Szymański & Kazdepka-Ziemińska 2003). Homocysteine accumulation also has detrimental effects on the vascular endothelium, leading to reduced NO synthesis and bioavailability (Stühlinger *et al.* 2001). NO plays a vital role as a paracrine mediator and modulator of ovarian functions, including ovulation, folliculogenesis, early embryonic cleavage, oocyte quality, implantation, uterine quiescence, endometrial receptivity, pregnancy, and uterine contractions and relaxation (Nath *et al.* 2019). Therefore, insufficient NO synthesis due to elevated homocysteine levels can have adverse reproductive outcomes. For example, studies with eNOS knockout mice have demonstrated significantly reduced ovulation rates, abnormal oocyte meiosis and increased oocyte mortality compared to normal mice, indicating that the lack of NO inhibits meiosis and oocyte maturation (Jablonka-Shariff & Olson 1997).

## Discussion and future perspectives

Reviewed studies have reported diminished ovarian reserve, ovarian sickling, gonadal hypo-function and increased incidence of maternal and perinatal complications that may lead to infertility and higher maternal mortality rates among women with SCD. The poor reproductive health outcomes in this population have been attributed in part to OS and imbalances in inflammation. Given the positive impact of B12 on female reproductive health, as well as its antioxidant and anti-inflammatory activities, B12 supplementation may help mitigate the deleterious effects of lipid peroxidation on the ovaries and regulate the production of ROS, thereby potentially improving the overall reproductive health of women with SCD.

The beneficial effects of B12 on the ovaries and in pregnancy may be attributed to its ability to decrease homocysteine accumulation, increase NO bioavailability, and reduce oxidative and inflammatory damage to the ovaries. While few studies have demonstrated the positive effects of B12 on female reproductive outcomes and during pregnancy, no such studies have been conducted specifically in SCD women. Therefore, there is a need for randomised controlled trials and prospective studies focusing specifically on this population to confirm the potential beneficial effects of B12. Such research will provide more empirical evidence to guide healthcare policies and highlight the urgent need to develop intervention strategies aimed at reducing poor reproductive health outcomes, pregnancy complications, and maternal and child mortality rates in this population. Additionally, multicentre studies with large sample populations in sub-Saharan Africa are required to evaluate the extent of B12 deficiency in individuals with SCD and develop suitable cost-effective treatment approaches.

The cellular deficiency of B12 in individuals with SCD is influenced by various factors. Along with conducting further studies, it is recommended to include routine assessment of B12 status in women living with SCD, especially in regions with a high burden of the disease like sub-Saharan Africa. This assessment aims to identify potential factors that could reduce B12 absorption and contribute to B12 deficiency in this population. By implementing routine B12 status evaluation, healthcare providers can better understand and address the specific needs of women with SCD, ultimately improving their overall health outcomes.

Considering the increased demand for B12 imposed by SCD, the current recommended dietary allowance (RDA) of 2.4 µg/day for adults may not be sufficient to maintain normal physiologic function, metabolism and prevention of subclinical B12 deficiency in this population. Particularly for reproductive-age women living with SCD, including those who are pregnant or lactating, a tailored dietary intake of B12 is necessary. To determine the optimal B12 dosage for individuals with SCD, an RCT comparing different doses of B12 could be conducted. Such a study would provide valuable insights and contribute to the development of specific RDAs for female patients with SCD, taking into account their unique nutritional requirements.

## Declaration of interest

The authors declare that there is no conflict of interest that could be perceived as prejudicing the impartiality of this review.

## Funding

This study did not receive any specific grant from any funding agency in thepublic, commercial or not-for-profit sector.

## Author contribution statement

TA conceived the idea, conducted the literature search and produced the initial draft of the manuscript. EA designed the figures. TA, EA and FR reviewed and edited the initial draft of the manuscript. All authors approved the final draft for submission.
